# Antimicrobial Efficacy of a Novel Antibiotic-Eluting Injectable Platelet-Rich Fibrin Scaffold against a Dual-Species Biofilm in an Infected Immature Root Canal Model

**DOI:** 10.1155/2020/6623830

**Published:** 2020-12-08

**Authors:** Azade Rafiee, Mahtab Memarpour, Yasaman Najibi, Bahman Khalvati, Sedigheh Kianpour, Mohammad Hossein Morowvat

**Affiliations:** ^1^Oral and Dental Disease Research Center, Department of Pediatric Dentistry, Dental School, Shiraz University of Medical Sciences, Shiraz, Iran; ^2^Student Research Committee, Dental School, Shiraz University of Medical Sciences, Shiraz, Iran; ^3^Medicinal Plants Research Center, Yasuj University of Medical Sciences, Yasuj, Iran; ^4^Department of Pharmaceutical Biotechnology, School of Pharmacy, Shiraz University of Medical Sciences, Shiraz, Iran; ^5^Pharmaceutical Sciences Research Center, Shiraz University of Medical Sciences, P.O. Box 71468-64685, Shiraz, Iran

## Abstract

**Background and Aims:**

This study was aimed at evaluating the antibacterial property of an injectable platelet-rich fibrin (I-PRF) scaffold containing triple antibiotic mixture against an *Actinomyces naeslundii* (*A. naeslundii*) and *Enterococcus faecalis* (*E. faecalis*) biofilm in an infected immature root canal model.

**Methods:**

A dual-species biofilm was inoculated inside the root canals via a series of centrifugal cycles. The samples were allocated to three experimental groups (i.e., G1: triple antibiotic mixture, G2: I-PRF containing triple antibiotic mixture, and G3: antibiotic-free I-PRF scaffold) and two control groups (G4: seven-day biofilm untreated and G5: bacteria-free untreated).

**Results:**

Bacterial gene quantification change and the overall reduction of live bacteria were evaluated. The highest antibacterial activity against *A. naeslundii* belonged to G2. However, G1 and G2 had similar antibacterial property against *E. faecalis* (*p* value = 0.814). In general, experimental groups revealed higher levels of antibacterial activity against *E. faecalis* than against *A. naeslundii* (*p* value < 0.001). Notably, G2 could dramatically decrease the number of live bacteria up to near 92%.

**Conclusions:**

The current study provides insight into the antibacterial property of an antibiotic-eluting I-PRF scaffold against a dual-species biofilm colonized inside the root canal. The fabricated scaffold contains not only the antibiotics but also the growth factors, which favor the regeneration.

## 1. Introduction

The symbiosis of various bacterial species, mostly facultative and strict anaerobic bacteria, inside the root canal system plays an essential role in endodontic infection [1]. Interactions among different bacterial cell proteins create a highly organized structure known as a biofilm [1]. Great variation in the composition of the microbiota inside the root canals has been identified including, but not limited to, *Enterococcus faecalis* (*E. faecalis*) and *Actinomyces naeslundii* (*A. naeslundii*) [2, 3]. *E. faecalis*, a gram-positive facultative anaerobe, is the main pathogen responsible for endodontic treatment failure [4]. Meanwhile, *A. naeslundii*, a gram-positive rod-shaped facultative anaerobe, is the most prevalent species in traumatized immature permanent teeth with necrotic pulps [5].

The regenerative endodontic procedure is an alternative to root apexification of a necrotic immature permanent tooth [6]. Briefly, copious chemical canal irrigation with minimal or no mechanical instrumentation, placement of an antibacterial medicament, removal of the medicament after nearly 1-4 weeks, and bleeding stimulation are the main steps in regenerative endodontics [7]. Complete elimination of the microbial population is one of the most crucial yet challenging steps in regenerative-based treatments [8]. Routinely, this is achieved by the intracanal placement of calcium hydroxide, triple antibiotic paste (TAP) containing ciprofloxacin (CIP), metronidazole (MET), and minocycline (MINO), or double antibiotic paste (DAP), containing CIP and MET (Kamocki et al., 2015). Generally, the use of either calcium hydroxide or antibiotic paste can cause unfavorable side effects. The former is responsible for root weakening even after a short period [9]. The latter has been proven to impair dental pulp stem cells when used at the clinically recommended dosage [3, 10].

In the light of this, recent researches have focused on the development of the local drug delivery scaffolds to both maximize root canal disinfection and reduce the risk of stem cell toxicity [2, 3, 9, 11–13]. Albuquerque et al. have conducted a series of investigations evaluating the antimicrobial effects of their developed antibiotic-containing electrospun scaffold against an *A. naeslundii*, *E. faecalis*, and multispecies biofilm model [2, 3, 11]. Their results revealed pronounced microbial biofilm elimination, a critical step in regenerative endodontics [2, 3, 11].

Bottino et al.'s researches have focused on the development of a cell-friendly scaffold capable of sufficient intracanal antibiotic delivery [12, 13]. As their results suggested, the synthesized antibiotic-containing electrospun scaffold could act as a biologically safe antimicrobial drug delivery system for regenerative endodontics [12, 13].

Among different generations of platelet concentrates, injectable platelet-rich fibrin (I-PRF) is one of the most recently introduced PRF clots. As the name suggests, it can be injected into the application site and form a small clot a few minutes after the injection. It contains higher concentrations of platelets, leukocytes, stem cells, and growth factors due to a decrease in centrifugation force and time [14]. While the regenerative potential of platelet concentrates is well studied, its antimicrobial properties have been reported in recent years [15].

Contemporary microbiological diagnostic techniques such as polymerase chain reaction (PCR) have been developed to improve the sensitivity and precision of specific pathogen detection and to overcome the drawbacks of microbiological culture methods [16]. The real-time PCR (RT-PCR) technique allows for monitoring and analysis of PCR product accumulation after each amplification cycle using fluorescent signals. Thereby, post-PCR processing and carryover contamination risk is eliminated [17]. SYBR Green I, which is the most routinely used to generate fluorescent signals proportional to the amount of a double-stranded DNA (dsDNA) PCR product, provides the ability to detect minute amounts of the target sequence, as well as the ability to quantify the target DNA [17].

The main disadvantage of PCR methods is the limitation of distinguishing between live and dead cells. In other words, a bacterial DNA sample can survive long after the bacterial cell death [18]. The MTT assay is a colorimetric assay for detecting metabolic activity. Enzymatic reduction of MTT (a yellow tetrazolium) to formazan crystal correlates with the bacterial viability or bacterial reproductive capacity [19].

Recently, our group has incorporated triple antibiotic mixture (1 mg/ml) into an I-PRF scaffold and has evaluated its drug release profile up to 28 days by using high-performance liquid chromatography (HPLC) [20]. To our knowledge, this is the first study to investigate the antimicrobial effects of the I-PRF scaffold containing triple antibiotic mixture for root canal disinfection. Therefore, this study was aimed at evaluating the antimicrobial profile of the newly developed antibiotic-containing I-PRF against a dual-species biofilm (*A. naeslundii* and *E. faecalis*) in an infected immature root canal model by using RT-PCR and the MTT assay.

## 2. Materials and Methods

### 2.1. Sample Preparation

Sixty-four intact, caries-free single-rooted human mandibular first premolars, which were extracted for orthodontic reasons, were collected based on a protocol approved by the Ethics Review Committee of Shiraz University of Medical Sciences (IR.SUMS.DENTAL.REC.1398.078). The collected tooth samples went through the process of washing, scaling the tissue tags, disinfecting in 0.9% (*w*/*v*) NaCl containing 0.02% sodium azide, and storing in weekly renewed distilled water at 4°C until use [21]. Then, the teeth were sectioned at or below the cementoenamel junction with a diamond disc to adjust 14 mm length. After access cavity preparation, the root canals were instrumented and widened to an internal diameter close to 2 mm under copious irrigation with 2 mL of 5.25% sodium hypochlorite and 5 mL of 17% EDTA, to simulate immature root apices [22]. Finally, the root canals were rinsed with deionized water and dried by using paper points. Light cure resin and two layers of nail polish on the external surface of teeth sealed the apex of all roots. All specimens were autoclaved at 121°C for 30 min for further use [21]. The sterility of external and internal surfaces was confirmed by the aerobic and anaerobic culturing methods.

### 2.2. Preparation of I-PRF and Triple Antibiotic Mixture

A stock solution of triple antibiotic mixture (Merck, Germany) was prepared by mixing 10 mg of MINO (CAS number 13614-98-7), MET (CAS number 443-48-1), and CIP (CAS number 85721-33-1) in 10 mL of deionized water reaching a final concentration of 1 mg/mL.

For I-PRF fabrication, the blood samples were obtained from members of our research team between the ages of 20 and 35 with the informed consent of volunteer donors. Next, tubes of 9 mL of whole blood without anticoagulant were immediately centrifuged at 60 g for 3 min at 4°C (Eppendorf, Westbury, NY centrifuge; Hamburg, Germany), and the upper liquid layer was collected as I-PRF.

After the preparation of I-PRF scaffolds, 2 mL of the collected I-PRF sample was transferred into each plastic microtube. Then, 2 mL of the stock solution of the triple antibiotic mixture was added to each microtube using a sampler (Eppendorf, Germany) while being horizontally shaken at 150 rpm (PECO, shaker-incubator, Iran).

### 2.3. Intracanal Dual-Species Biofilm Formation

Fifty-six sterile tooth samples, randomly placed inside microcentrifuge tubes containing 300 mL suspension of *A. naeslundii* (PTCC 1201) and 300 mL suspension of *E. faecalis* (PTCC 1778), followed a sequence of centrifugal cycles (2 each) at 1400 g, 2000 g, 3600 g, and 5600 g for 5 minutes for bacterial penetration. The bacterial suspension was refreshed between each cycle. The infected teeth were incubated under microaerophilic conditions (Anoxomat System AN2OP, MART Microbiology B.V., Netherlands) (37°C, 5.8% O_2_) in microtubes containing 1 mL fresh and sterile brain-heart infusion (BHI)+1% sucrose for seven days to allow for biofilm formation. The broth was replaced every other day to preserve bacterial viability [11]. Eight samples remained bacteria-free as the negative control.

### 2.4. Sampling Procedure and Group Allocation

After biofilm formation, the infected (*n* = 56) and uninfected (*n* = 8) samples were subjected to the first sampling procedure (S1 sample). Briefly, each tooth was held with a sterile clamp, and the external surface was disinfected with 30% hydrogen peroxide followed by 2.5% NaOCl and inactivated with 5% sodium thiosulfate. Each root canal was gently rinsed with 1 mL of BHI to remove unattached bacteria and was instrumented by using 30 strokes of a sterile 35-H file [23]. Sterile paper points were placed into each root canal to achieve dryness. Afterwards, the paper points were transferred in a sterile microtube containing 2 mL BHI, frozen at −20°C, for further use [21].

**Figure 1:**
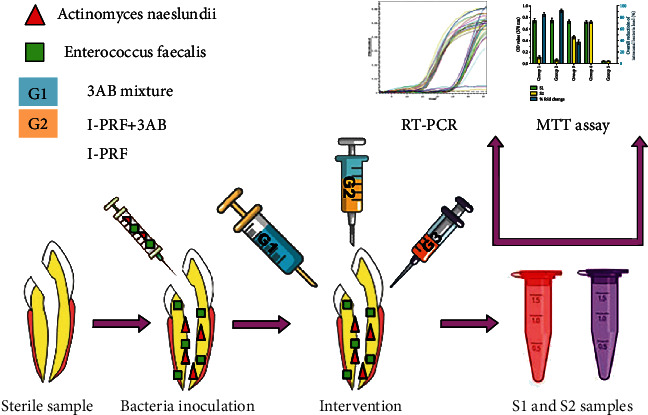
A brief overview on the exploited group allocation and sampling procedure in the current study. G1 represents the triple antibiotic mixture, G2 is the I-PRF containing triple antibiotic mixture, and G3 means the antibiotic-free I-PRF scaffold.

After S1 sampling (Figure 1), the tooth samples were randomly allocated into three experimental groups (*n* = 16/group) (G1: triple antibiotic mixture (1 mg/mL), G2: I-PRF containing triple antibiotic mixture, and G3: antibiotic-free I-PRF scaffold) and two control groups (*n* = 8/group) (G4: seven-day biofilm untreated and G5: bacteria-free untreated). Antibiotic mixture, I-PRF, antibiotic-containing I-PRF scaffold, or BHI for each group was injected with the sterile insulin syringe.

Next, the coronal seal was provided for all samples by using a light cure glass ionomer (GC, Japan). All samples were preserved in artificial saliva for seven days under the microaerophilic condition at 37°C while being horizontally shaken at 150 rpm (PECO, shaker-incubator, Iran) to maintain a humid environment and to simulate the oral cavity condition.

The second sampling (S2 samples) was performed for all groups after the explained intervention, as mentioned previously. After field disinfection and before complete removal of the coronal seal, a new sterile bur without the use of water spray was replaced. The intracanal medicaments were washed thrice with 1 mL sterile PBS solution to remove the loosely bound bacteria and the residual antiseptics of the root canal walls [19].

### 2.5. Microbiological Assays

The collected S1 and S2 samples in each group were divided into two equal subgroups for further microbiological assays using RT-PCR and the MTT assay.

### 2.6. DNA Extraction, RT-PCR Conditions, and Primer Design

After thawing the frozen paper point samples, the tubes were vortexed for 60 s at 3000 rpm (micro-spin fuge/vortex, Kiagen, Iran) and centrifuged for 10 min at 10000 rpm (Eppendorf, Westbury, NY centrifuge; Hamburg, Germany) to separate the absorbed microbial cells from the paper points. Thereafter, the supernatant was discarded, and the bacterial precipitation was used for the next step [24]. The genomic DNAs were extracted using the One-Tube Bacterial Genomic DNA Extraction Kit (Bio Basic Inc., Canada) according to the manufacturer's instructions and were stored at −20°C until they were ready for use as templates for PCR amplification.

The sequence of forward and reverse primers for *E. faecalis* and *A. naeslundii*, as well as the primer sequence specific to 16S rDNA selected as an endogenous control for the study, is listed in Table 1.

Real-time PCR analysis was performed by the iCycler iQ real-time detection system (Bio-Rad Laboratories). Real-time PCR reaction mixture in 10 *μ*L total volume contained 2.3 *μ*L of sterile distilled water, 0.6 *μ*L (0.5 *μ*M) of each primer (reverse and forward), 5 *μ*L of Universal Master Mix (RealQ Plus Master Mix Green A343202, Ampliqon, Denmark), and 1.5 *μ*L (10 ng) of template DNA.

**Figure 2:**
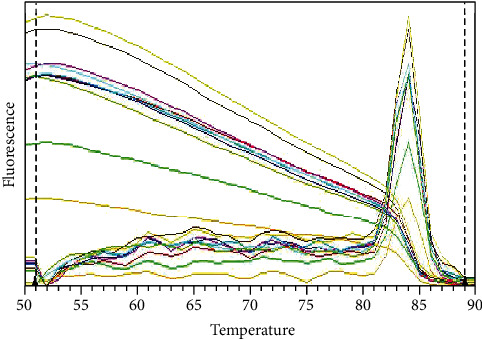
The determined melting curve in RT-PCR analysis using SYBR green fluorescence between 50°C and 90°C.

The amplification cycles involved initial template denaturation at 95°C for 15 min, followed by 35 cycles of denaturation at 95°C for 15 s, annealing at 60°C for 30 s, and elongation at 72°C for 30 s. A melting curve was determined using SYBR green fluorescence between 50°C and 90°C (Figure 2), with a ramp speed of 0.2°C/s and reading at every 0.2°C [23]. Cycle threshold (Ct) values in PCR reactions were analyzed, and an automatic threshold setting of 0.2 was used for all samples.

### 2.7. Evaluation of Bacterial Metabolic Activity (MTT Assay)

The overall reduction in viable bacterial load along the canal walls was assessed for each experimental group (*n* = 8) and the control groups (*n* = 4) using the MTT assay. The tubes containing S1 and S2 samples were gently vortexed for 1 min and incubated at 37°C for 4 hours. After adding 200 *μ*L of MTT solution (5 mg/mL) to each tube, the mixture was incubated at 37°C for 4 h for the conversion of MTT to formazan crystal. Dimethyl sulfoxide was added to each tube to solubilize formazan crystals. After shaking for 15 min, optical density of the experimental groups and the blank mixture containing 2 mL BHI, 200 *μ*L MTT, and 2 mL dimethyl sulfoxide was recorded at 570 nm using an Epoch microplate spectrophotometer (Epoch, BioTek Instruments, Winooski, VT, USA) [21].

### 2.8. Statistical Analysis

All measurements, made in triplicate, are reported as mean value ± the standard deviation (±SD) of the mean using SPSS version 22.0 (IBM Corp., USA) software. The delta-delta Ct method (2^–*ΔΔ*Ct^ method) was used to calculate the relative fold gene quantification of RT-PCR samples, as shown in the following equations:

ΔCt = Ct (gene of interest)–Ct (housekeeping gene); ΔΔCt = ΔCt (S2)–ΔCt (S1); Fold gene quantification = 2^‐(ΔΔCt)^

The calculated values (2^-(*ΔΔ*Ct)^) were log_10_-transformed to normalize the data before analysis [25]. Comparison of antimicrobial activity of the interventional groups against each bacterial species was performed using one-way ANOVA, followed by a post hoc test (Tukey test). Comparison of each bacterial species fold gene quantification change following each intervention was performed using the *t*-test. The bacterial reduction from S1 to S2 was compared by using the paired *t*-test.

For the MTT assay, the percent bacterial reduction was calculated using the following formula [26]:

Percent bacterial reduction = (S1 − S2/S1) × 100.

The results were used to compare the overall differences using one-way ANOVA, followed by the Tukey post hoc test. The statistical level of significance was set at *p* < 0.05.

## 3. Results and Discussion

According to the independent paired *t*-test, all experimental groups revealed a significant decrease in fold gene quantification values after the intervention (*p* value < 0.001).

Comparison of the calculated logarithmic values of DNA load for each bacterial species using one-way ANOVA and the Tukey post hoc test showed significant differences in the antimicrobial properties of the experimental groups (*p* value < 0.001). Tables 2 and 3 demonstrate the mean differences between G1, G2, and G3 antimicrobial activities against *A. naeslundii* and *E. faecalis*. As shown in Table 2, G2 showed the highest *A. naeslundii* gene quantification reduction, followed by G1 and G3. However, G1 and G2 did not represent noticeable differences in antibacterial effect against *E. faecalis* (*p* value = 0.814) (Table 3). Likewise, G3 exhibited the least antibacterial activity against *E. faecalis* (*p* value < 0.001).

Comparison of each bacterial species fold gene quantification change following each intervention using the *t*-test showed remarkably higher antibacterial property against *E. faecalis* than against *A. naeslundii* (*p* value < 0.001) (Table 4).

**Figure 3:**
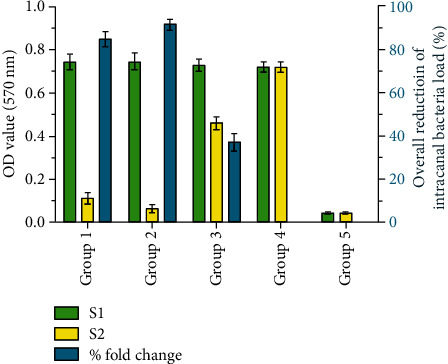
Bacterial viability and the overall percent fold change reduction based on differences in bacterial metabolic activity (MTT assay) before (S1) and after (S2) the intervention.

Bacterial viability from the S1 and S2 sample collection was investigated using the MTT assay, as shown in Figure 3. Comparison of the fold change reduction using one-way ANOVA and the Tukey post hoc test illustrated remarkable differences between bacterial metabolic activities in each group (*p* value < 0.001). The maximum and minimum bacterial reduction efficiency belonged to G2 and G3, respectively.

Local delivery of the endodontic medicaments has become a crucial area for intracanal disinfection [27]. Recently, our group has demonstrated the drug release profile of a novel antibiotic-eluting I-PRF-based scaffold by using HPLC [20]. Our newly developed scaffold held promises as an intracanal medicament to subsequently release the absorbed antibiotics over 28 days [20]. As far as we know, this is the first study to investigate the antimicrobial profile of the antibiotic-containing I-PRF against a dual-species biofilm (*A. naeslundii* and *E. faecalis*) in an infected immature root canal model.

Both *E. faecalis* and *A*. *naeslundii* have been correlated with the capacity of biofilm formation and dentinal tubule invasion [3, 21]. In this way, these two bacterial species (*E. faecalis* and *A. naeslundii*) were selected for the present study. Most of the studies have developed a single-species (mainly *E. faecalis*) biofilm for antimicrobial assessments [2, 3, 28]. However, a limited number of studies have reported on the development of a multispecies biofilm on human dentin [11, 19, 29]. Here, we developed a young, 7-day-old dual-species biofilm on human dentin. Importantly, a sequence of centrifugal cycles of dentin specimens in microtubes containing bacterial suspension permitted dentinal tubule invasion, simulating long-term infection [30, 31]. In our study, the root canals were further widened to an internal diameter close to 2 mm. This sample model had the benefits of both immature root canal imitation [22] and intracanal antibacterial activity assessment [30].

Real-time PCR technology provides the possibility of absolute or relative sample quantification analysis [32]. Absolute quantification determines the amount of a target sequence by back-calculation from the standard curve. In contrast, relative quantification relates the PCR signal of the target DNA in a sample, such as the experimental group, to that of another sample, such as an untreated control. The delta-delta Ct method (2^–*ΔΔ*Ct^ method) is a convenient way for relative quantification [25]. The 16S rDNA nucleotide sequence has been generally used as a housekeeping gene to detect bacterial species, because they are well conserved [16].

From an antimicrobial viewpoint, a significant reduction in the fold gene change was observed in all experimental groups (*p* value < 0.001). The highest antimicrobial activity against *A. naeslundii* was found in G2, while G1 and G2 demonstrated no significant difference in antibacterial properties against *E. faecalis* (*p* value = 0.814). These findings are in line with the results of microbial biofilm elimination capacity of synthetic scaffolds capable of intracanal antibiotic delivery [2, 3, 11–13]. *E. faecalis* have characteristics to survive as a biofilm in extreme environmental challenges as well as to invade and proliferate within the dentinal tubules up to 1000 *μ*m [21]. Despite a considerable reduction in the population of both bacteria, our fabricated scaffold showed remarkably higher antibacterial property against *E. faecalis* than against *A. naeslundii* (*p* value < 0.001) (Table 4).

DNA-based techniques have limitation for the detection of the live bacterial population, because bacterial DNA released from dead cells might still be detectable [16]. Therefore, we implemented the MTT assay for bacterial viability assessment. Notably, G2 and G1 could dramatically decrease the number of live bacteria up to 91.62 ± 2.069% and 84.922 ± 3.107%, respectively (Figure 3). The maximum and minimum bacterial reduction efficiency belonged to G2 and G3, respectively. Interestingly, G3 could reduce the number of live bacteria to near 37%. The mechanisms underlying the antimicrobial activity of I-PRF are not fully known. It seems that the complex mixture of platelets, plasmatic component (such as the complement system), and leukocytes is mainly responsible for its antimicrobial profile [15].

Over the last few years, TAP has been widely used as an intracanal medicament for disinfection purposes within the regenerative approach. CIP is a member of the fluoroquinolone class of antibiotics with bactericidal activity. It prevents the DNA replications in bacteria (routinely gram-negative bacilli) through inhibiting two major bacterial enzymes: DNA topoisomerase and DNA gyrase. MET, a nitroimidazole antibiotic, is mainly used against anaerobic and microaerophilic bacterial infections. The mechanisms of bacterial cell death are interacting with DNA, losing the helical DNA structure, breaking the strands, and finally inhibiting the protein synthesis. MINO is a second generation of tetracycline antibiotics with a wide-spectrum activity against gram-negative and gram-positive infections. Its antibacterial activity involves its attachment to the 30S ribosomal subunit and prevention of the protein synthesis procedure. Meanwhile, biodegradable and compatible polymer nanofibers with considerably lower antibiotic concentration have shown promising bacterial killing in multispecies models [2, 11]. The critical concerns towards the intracanal use of scaffolds relate to the attainment of an intimate contact within the dentinal wall, delivery of sufficient amounts of antimicrobials followed by their sustained maintenance, and consequently avoiding the stem cell toxicity [2]. It is worth emphasizing that the significantly low and clinically safe concentration of antibiotics [33] within our developed I-PRF construct (1 mg/scaffold), contrary to the pasty consistency (1 g/mL), highlights its suitability as an antimicrobial effective intracanal local drug delivery system.

We recommend further studies to explore the tooth discoloration potential and the possibility of antibiotic resistance of intracanal bacteria. Moreover, animal studies are suggested for future human application. This approach might open a new way to one-session regenerative treatment modalities.

## 4. Conclusion

Taken together, the fabricated scaffold could dramatically reduce both the total bacterial gene quantification and the number of live bacteria inside the root canal. These findings further emphasize the promising potential of an antibiotic-eluting I-PRF scaffold for disinfection of an immature root canal model with a dual-species biofilm (*E. faecalis* and *A. naeslundii*).

## Figures and Tables

**Table 1:** List of the employed forward and reverse primers for *E. faecalis* and *A. naeslundii*, as well as the 16S rDNA amplification as the endogenous control in the current study.

Bacterial name	Forward primer	Reverse primer	Size of amplification	Reference
*A. naeslundii*	CTGCTGCTGACATCGCCGCTCGTA	TCCGCTCGCGCCACCTCTCGTTA	144	[34]
*E. faecalis*	CCGAGTGCTTGCACTCAATTGG	CTCTTATGCCATGCGGCATAAAC	138	[35]
16S rDNA	TTAAACTCAAAGGAATTGACGG	CTCACGACACGAGCTGACGAC	170	[35]

**Table 2:** The mean differences between the antimicrobial activities of experimental groups against *A. naeslundii*. G1 represents the triple antibiotic mixture, G2 is the I-PRF containing triple antibiotic mixture, and G3 means the antibiotic-free I-PRF scaffold.

Bacterial species	Group (I)		Group (J)	Mean differences (I–J) ± SD	*p* value
	Group number	Mean ± SD	Group number		
*A. naeslundii*	G1	1.24 ± 0.115	G2	−1.27 ± 0.07	≤0.001^∗^
			G3	0.26 ± 0.07	0.004^∗^
	G2	2.51 ± 0.141	G1	1.27 ± 0.07	≤0.001^∗^
			G3	1.53 ± 0.07	≤0.001^∗^
	G3	0.98 ± 0.171	G1	−0.26 ± 0.07	0.004^∗^
			G2	1.53 ± 0.07	≤0.001^∗^
Total	1.575 ± 0.6969				≤0.001^∗^

SD: standard deviation. ^∗^Significant difference (*p* value < 0.05).

**Table 3:** The mean differences between the antimicrobial activities of experimental groups against *E. faecalis*. G1 represents the triple antibiotic mixture, G2 is the I-PRF containing triple antibiotic mixture, and G3 means the antibiotic-free I-PRF scaffold.

Bacterial species	Group (I)		Group (J)	Mean differences (I–J) ± SD	*p* value
	Group number	Mean ± SD	Group number		
*E. faecalis*	G1	3.364 ± 0.124	G2	−0.033 ± 0.054	0.814
			G3	0.923 ± 0.054	≤0.001^∗^
	G2	3.4 ± 0.091	G1	0.033 ± 0.054	0.814
			G3	0.956 ± 0.054	≤0.001^∗^
	G3	2.441 ± 0.105	G1	−0.923 ± 0.054	0.004^∗^
			G2	−0.956 ± 0.054	≤0.001^∗^
Total	3.067 ± 0.464				≤0.001^∗^

SD: standard deviation. ^∗^Significant difference (*p* value < 0.05).

**Table 4:** Comparison of the observed fold gene quantification changes in *A. naeslundii* and *E. faecalis*. G1 represents the triple antibiotic mixture, G2 is the I-PRF containing triple antibiotic mixture, and G3 means the antibiotic-free I-PRF scaffold.

Group number	*A. naeslundii* (mean ± SD)	*E. faecalis* (mean ± SD)	Mean differences ± SD	*p* value
1	1.24 ± 0.115	3.364 ± 0.124	−2.125 ± 0.184	≤0.001^∗^
2	2.51 ± 0.141	3.4 ± 0.091	−0.888 ± 0.213	≤0.001^∗^
3	0.98 ± 0.171	2.441 ± 0.105	−1.463 ± 0.155	≤0.001^∗^

SD: standard deviation. ^∗^Significant difference (*p* value < 0.05).

## Data Availability

All generated or analyzed data and the exploited software and materials were included in this published article. The generated results during the current study are available from the corresponding author on reasonable request.
